# 1,1′-(Phenyl­methyl­ene)dinaphthalen-2-ol

**DOI:** 10.1107/S1600536812004163

**Published:** 2012-02-04

**Authors:** Wen-Ni Zheng

**Affiliations:** a College of Chemistry and Chemical Engineering, Southeast University, Nanjing 210096, People’s Republic of China

## Abstract

In the title compound, C_27_H_20_O_2_, the phenyl ring is oriented with respect to the naphthalene ring systems at 57.87 (6) and 85.12 (6)°. The two naphthalene ring systems make a dihedral angle of 70.10 (4)°. In the mol­ecule, the hy­droxy groups are involved in a strong intra­molecular O—H⋯O hydrogen bond. In the crystal, inversion dimers linked by pairs of O—H⋯O hydrogen bonds occur. A weak C—H⋯π inter­action is also observed in the crystal.

## Related literature

For the structures and ferroelectric properties of related compounds, see: Devi & Bhuyan (2004 ▶); Fu, Zhang, Cai, Ge *et al.* (2011 ▶); Fu, Zhang, Cai, Zhang, Ge, Xiong & Huang (2011 ▶); Fu, Zhang, Cai, Zhang, Ge, Xiong, Huang & Nakamura (2011 ▶); Fu *et al.* (2007 ▶, 2008 ▶, 2009 ▶); Fu & Xiong (2008 ▶).
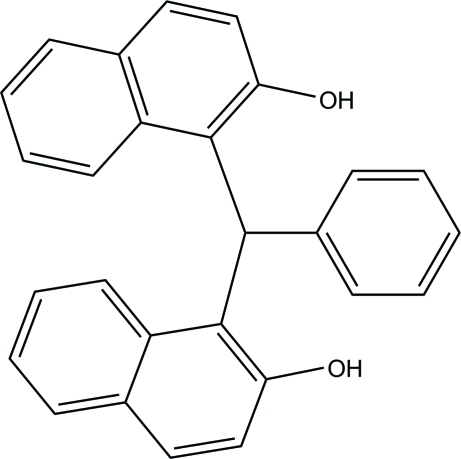


## Experimental

### 

#### Crystal data

C_27_H_20_O_2_
*M* *_r_* = 376.43
Monoclinic, 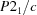

*a* = 12.066 (2) Å
*b* = 8.6178 (17) Å
*c* = 21.386 (6) Å
β = 122.02 (2)°
*V* = 1885.4 (7) Å^3^
*Z* = 4
Mo *K*α radiation
μ = 0.08 mm^−1^
*T* = 298 K
0.10 × 0.03 × 0.03 mm

#### Data collection

Rigaku Mercury2 (2 × 2 bin mode) diffractometer
Absorption correction: multi-scan (*CrystalClear*; Rigaku, 2005 ▶) *T*_min_ = 0.910, *T*_max_ = 1.000
19010 measured reflections
4317 independent reflections
2997 reflections with *I* > 2σ(*I*)
*R*_int_ = 0.063

#### Refinement

*R*[*F* ^2^ > 2σ(*F* ^2^)] = 0.056
*wR*(*F* ^2^) = 0.142
*S* = 1.06
4317 reflections
262 parameters
H-atom parameters constrained
Δρ_max_ = 0.40 e Å^−3^
Δρ_min_ = −0.42 e Å^−3^

### 

Data collection: *CrystalClear* (Rigaku, 2005 ▶); cell refinement: *CrystalClear*; data reduction: *CrystalClear*; program(s) used to solve structure: *SHELXS97* (Sheldrick, 2008 ▶); program(s) used to refine structure: *SHELXL97* (Sheldrick, 2008 ▶); molecular graphics: *SHELXTL* (Sheldrick, 2008 ▶); software used to prepare material for publication: *SHELXTL*.

## Supplementary Material

Crystal structure: contains datablock(s) I, global. DOI: 10.1107/S1600536812004163/xu5462sup1.cif

Structure factors: contains datablock(s) I. DOI: 10.1107/S1600536812004163/xu5462Isup2.hkl

Supplementary material file. DOI: 10.1107/S1600536812004163/xu5462Isup3.cml

Additional supplementary materials:  crystallographic information; 3D view; checkCIF report

## Figures and Tables

**Table 1:** Hydrogen-bond geometry (Å, °) *Cg* is the centroid of the C22–C27 ring.

*D*—H⋯*A*	*D*—H	H⋯*A*	*D*⋯*A*	*D*—H⋯*A*
O1—H1⋯O2^i^	0.93	2.49	3.335 (2)	151
O2—H2⋯O1	0.86	1.85	2.691 (2)	165
C19—H19⋯*Cg*^ii^	0.93	2.71	3.478 (3)	140

Symmetry codes: (i) 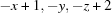; (ii) 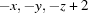.

**Fig. 1.**
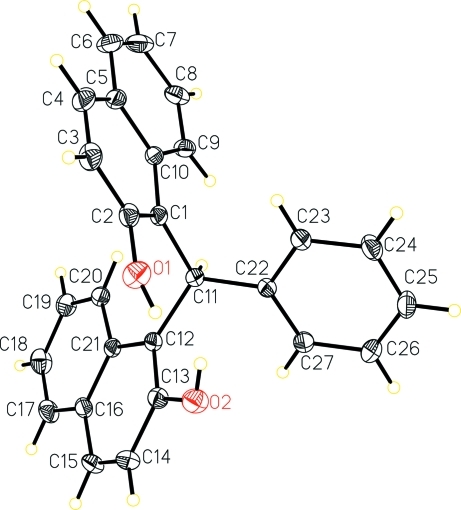
A view of the asymmetric unit with the atomic numbering scheme. The displacement ellipsoids were drawn at the 30% probability level.

## supplementary crystallographic information

### Comment

Simple organic compounds containing strong intramolecular H-bonds have attracted an attention as materials which display ferroelectric-paraelectric phase transitions (Fu, Zhang, Cai, Ge *et al.*, 2011; Fu, Zhang, Cai, Zhang, Ge, Xiong & Huang, 2011; Fu, Zhang, Cai, Zhang, Ge, Xiong, Huang & Nakamura, 2011). With the purpose of obtaining phase transition crystals of organic compounds, various organic molecules have been studied and a series of new materials have been elaborated (Fu *et al.* 2007; Fu & Xiong 2008; Fu *et al.* 2008; Fu *et al.* 2009). Herewith we present the synthesis and crystal structure of the title compound, 1,1'-(phenylmethylene)dinaphthalen-2-ol.

In the title compound (Fig. 1) bond lengths and angles have normal values (Devi & Bhuyan 2004). The dihedral angle between the naphthalene ring systemes and the benzene ring are 57.87 (6)° and 85.12 (6)°, respectively. The H atoms of hydroxy groups were involved in intramolecular O—H···O hydrogen bonds. The weak intermolecular C—H···π interaction is present in the crystal structure with the C19···*Cg* = 3.478 (2)Å (*Cg* is the centroid of the C22 to C27 benzene ring) (Table 1).

### Experimental

A dry 50 ml flask was charged with benzaldehyde (10 mmol) and naphthalen-2-ol (20 mmol). The mixture was stirred at 373 K for 12 h and then added ethanol (15 ml), after heated under reflux for 1 h, the precipitate was filtrated out and washed with ethanol for 3 times to give the title compound. Colourless crystals suitable for X-ray diffraction were obtained by slow evaporation of a dichloromethane solution.

### Refinement

hydroxy H atoms were placed in chemical sensible positions and refined in a riding mode with U_iso_(H) = 1.5U_eq_(O). Other H atoms were situated into the idealized positions and treated as riding with C–H = 0.93 Å (aromatic) and 0.98 Å (methine), *U_iso_*(H) = 1.2*U_eq_*(C).

### Crystal data

**Table:**

C_27_H_20_O_2_	*F*(000) = 792
*M**_r_* = 376.43	*D*_x_ = 1.326 Mg m^−^^3^
Monoclinic, *P*2_1_/*c*	Mo *K*α radiation, λ = 0.71073 Å
Hall symbol: -P 2ybc	Cell parameters from 4317 reflections
*a* = 12.066 (2) Å	θ = 3.1–27.5°
*b* = 8.6178 (17) Å	µ = 0.08 mm^−^^1^
*c* = 21.386 (6) Å	*T* = 298 K
β = 122.02 (2)°	Block, colourless
*V* = 1885.4 (7) Å^3^	0.10 × 0.03 × 0.03 mm
*Z* = 4	

### Data collection

**Table:**

Rigaku Mercury2 (2x2 bin mode) diffractometer	4317 independent reflections
Radiation source: fine-focus sealed tube	2997 reflections with *I* > 2σ(*I*)
Graphite monochromator	*R*_int_ = 0.063
Detector resolution: 13.6612 pixels mm^-1^	θ_max_ = 27.5°, θ_min_ = 3.1°
CCD profile fitting scans	*h* = −15→15
Absorption correction: multi-scan (*CrystalClear*; Rigaku, 2005)	*k* = −11→11
*T*_min_ = 0.910, *T*_max_ = 1.000	*l* = −27→27
19010 measured reflections	

### Refinement

**Table:**

Refinement on *F*^2^	Primary atom site location: structure-invariant direct methods
Least-squares matrix: full	Secondary atom site location: difference Fourier map
*R*[*F*^2^ > 2σ(*F*^2^)] = 0.056	Hydrogen site location: inferred from neighbouring sites
*wR*(*F*^2^) = 0.142	H-atom parameters constrained
*S* = 1.06	*w* = 1/[σ^2^(*F*_o_^2^) + (0.0564*P*)^2^ + 0.4682*P*] where *P* = (*F*_o_^2^ + 2*F*_c_^2^)/3
4317 reflections	(Δ/σ)_max_ = 0.001
262 parameters	Δρ_max_ = 0.40 e Å^−^^3^
0 restraints	Δρ_min_ = −0.42 e Å^−^^3^

### Special details

**Table:**

Geometry. All esds (except the esd in the dihedral angle between two l.s. planes) are estimated using the full covariance matrix. The cell esds are taken into account individually in the estimation of esds in distances, angles and torsion angles; correlations between esds in cell parameters are only used when they are defined by crystal symmetry. An approximate (isotropic) treatment of cell esds is used for estimating esds involving l.s. planes.
Refinement. Refinement of F^2^ against ALL reflections. The weighted R-factor wR and goodness of fit S are based on F^2^, conventional R-factors R are based on F, with F set to zero for negative F^2^. The threshold expression of F^2^ > 2sigma(F^2^) is used only for calculating R-factors(gt) etc. and is not relevant to the choice of reflections for refinement. R-factors based on F^2^ are statistically about twice as large as those based on F, and R- factors based on ALL data will be even larger.

### Fractional atomic coordinates and isotropic or equivalent isotropic displacement parameters (Å^2^)

**Table:**

	*x*	*y*	*z*	*U*_iso_*/*U*_eq_	
C1	0.19967 (17)	0.11950 (19)	0.93766 (9)	0.0305 (4)	
C2	0.30035 (19)	0.1805 (2)	0.93322 (11)	0.0368 (4)	
C3	0.2816 (2)	0.2632 (2)	0.87169 (12)	0.0482 (5)	
H3	0.3531	0.3020	0.8713	0.058*	
C4	0.1591 (2)	0.2861 (3)	0.81308 (12)	0.0514 (6)	
H4	0.1466	0.3437	0.7731	0.062*	
C5	0.0501 (2)	0.2236 (2)	0.81202 (10)	0.0419 (5)	
C6	−0.0781 (3)	0.2448 (3)	0.75021 (12)	0.0569 (6)	
H6	−0.0903	0.3033	0.7105	0.068*	
C7	−0.1830 (2)	0.1820 (3)	0.74762 (11)	0.0568 (6)	
H7	−0.2667	0.1999	0.7072	0.068*	
C8	−0.1651 (2)	0.0898 (3)	0.80619 (11)	0.0466 (5)	
H8	−0.2370	0.0435	0.8036	0.056*	
C9	−0.04327 (19)	0.0671 (2)	0.86700 (10)	0.0389 (5)	
H9	−0.0340	0.0049	0.9050	0.047*	
C10	0.06952 (18)	0.1356 (2)	0.87383 (9)	0.0327 (4)	
C11	0.22543 (16)	0.03527 (19)	1.00723 (9)	0.0283 (4)	
H11	0.1378	0.0207	0.9986	0.034*	
C12	0.29752 (17)	0.1300 (2)	1.07956 (9)	0.0302 (4)	
C13	0.42859 (17)	0.1136 (2)	1.13304 (10)	0.0354 (4)	
C14	0.4834 (2)	0.1812 (2)	1.20371 (10)	0.0444 (5)	
H14	0.5711	0.1644	1.2392	0.053*	
C15	0.4102 (2)	0.2700 (2)	1.22052 (11)	0.0444 (5)	
H15	0.4473	0.3108	1.2678	0.053*	
C16	0.27814 (19)	0.3010 (2)	1.16688 (10)	0.0361 (4)	
C17	0.2019 (2)	0.3980 (2)	1.18281 (12)	0.0465 (5)	
H17	0.2396	0.4418	1.2295	0.056*	
C18	0.0746 (2)	0.4288 (3)	1.13131 (12)	0.0507 (6)	
H18	0.0254	0.4918	1.1429	0.061*	
C19	0.0184 (2)	0.3647 (2)	1.06077 (12)	0.0437 (5)	
H19	−0.0684	0.3866	1.0253	0.052*	
C20	0.08900 (18)	0.2701 (2)	1.04312 (10)	0.0347 (4)	
H20	0.0494	0.2297	0.9957	0.042*	
C21	0.22122 (17)	0.23241 (19)	1.09552 (9)	0.0303 (4)	
C22	0.27635 (16)	−0.1314 (2)	1.01420 (9)	0.0294 (4)	
C23	0.28219 (18)	−0.2027 (2)	0.95793 (10)	0.0351 (4)	
H23	0.2620	−0.1463	0.9161	0.042*	
C24	0.3181 (2)	−0.3583 (2)	0.96341 (12)	0.0453 (5)	
H24	0.3204	−0.4050	0.9249	0.054*	
C25	0.3501 (2)	−0.4432 (2)	1.02521 (12)	0.0484 (5)	
H25	0.3753	−0.5465	1.0290	0.058*	
C26	0.3445 (2)	−0.3740 (2)	1.08149 (12)	0.0475 (5)	
H26	0.3658	−0.4307	1.1234	0.057*	
C27	0.3074 (2)	−0.2203 (2)	1.07578 (10)	0.0402 (5)	
H27	0.3031	−0.1752	1.1140	0.048*	
O1	0.42620 (13)	0.15738 (17)	0.99198 (8)	0.0499 (4)	
H1	0.4672	0.1320	0.9670	0.075*	
O2	0.51565 (12)	0.02732 (16)	1.12482 (7)	0.0460 (4)	
H2	0.4961	0.0568	1.0819	0.069*	

### Atomic displacement parameters (Å^2^)

**Table:**

	*U*^11^	*U*^22^	*U*^33^	*U*^12^	*U*^13^	*U*^23^
C1	0.0414 (10)	0.0237 (8)	0.0308 (9)	0.0014 (7)	0.0222 (8)	−0.0012 (7)
C2	0.0456 (11)	0.0305 (9)	0.0414 (11)	0.0003 (8)	0.0279 (10)	−0.0019 (8)
C3	0.0693 (15)	0.0397 (12)	0.0542 (13)	−0.0073 (10)	0.0454 (13)	−0.0003 (10)
C4	0.0827 (17)	0.0401 (12)	0.0431 (12)	0.0011 (11)	0.0412 (13)	0.0074 (10)
C5	0.0634 (13)	0.0340 (10)	0.0317 (10)	0.0081 (9)	0.0274 (10)	0.0015 (8)
C6	0.0753 (17)	0.0580 (15)	0.0297 (11)	0.0189 (13)	0.0226 (12)	0.0096 (10)
C7	0.0533 (14)	0.0715 (16)	0.0290 (11)	0.0184 (12)	0.0105 (10)	−0.0018 (11)
C8	0.0426 (11)	0.0582 (13)	0.0333 (11)	0.0039 (10)	0.0162 (9)	−0.0108 (10)
C9	0.0433 (11)	0.0429 (11)	0.0280 (9)	0.0030 (9)	0.0173 (9)	−0.0009 (8)
C10	0.0443 (11)	0.0271 (9)	0.0275 (9)	0.0066 (8)	0.0196 (8)	−0.0015 (7)
C11	0.0296 (9)	0.0277 (9)	0.0277 (9)	−0.0001 (7)	0.0152 (8)	0.0000 (7)
C12	0.0355 (10)	0.0264 (9)	0.0287 (9)	−0.0024 (7)	0.0170 (8)	−0.0004 (7)
C13	0.0334 (10)	0.0337 (10)	0.0351 (10)	−0.0006 (8)	0.0155 (8)	−0.0007 (8)
C14	0.0385 (11)	0.0471 (12)	0.0332 (10)	−0.0039 (9)	0.0093 (9)	−0.0028 (9)
C15	0.0515 (13)	0.0429 (12)	0.0295 (10)	−0.0084 (9)	0.0152 (10)	−0.0102 (9)
C16	0.0487 (11)	0.0281 (9)	0.0334 (10)	−0.0058 (8)	0.0231 (9)	−0.0035 (8)
C17	0.0659 (15)	0.0395 (11)	0.0441 (12)	−0.0063 (10)	0.0360 (11)	−0.0115 (9)
C18	0.0610 (14)	0.0445 (12)	0.0611 (14)	0.0049 (10)	0.0421 (13)	−0.0086 (11)
C19	0.0437 (11)	0.0408 (11)	0.0500 (12)	0.0047 (9)	0.0271 (10)	0.0020 (10)
C20	0.0392 (10)	0.0316 (9)	0.0333 (10)	0.0015 (8)	0.0193 (9)	0.0012 (8)
C21	0.0373 (10)	0.0252 (9)	0.0307 (9)	−0.0029 (7)	0.0196 (8)	0.0006 (7)
C22	0.0288 (9)	0.0277 (9)	0.0289 (9)	−0.0015 (7)	0.0133 (7)	−0.0014 (7)
C23	0.0395 (10)	0.0323 (10)	0.0331 (9)	−0.0019 (8)	0.0189 (8)	−0.0029 (8)
C24	0.0576 (13)	0.0359 (11)	0.0475 (12)	−0.0006 (9)	0.0315 (11)	−0.0102 (9)
C25	0.0534 (13)	0.0270 (10)	0.0588 (14)	0.0034 (9)	0.0256 (11)	−0.0006 (10)
C26	0.0599 (14)	0.0343 (11)	0.0427 (12)	0.0020 (10)	0.0233 (11)	0.0090 (9)
C27	0.0543 (12)	0.0340 (10)	0.0333 (10)	−0.0010 (9)	0.0239 (10)	−0.0001 (8)
O1	0.0423 (8)	0.0567 (9)	0.0561 (9)	−0.0003 (7)	0.0299 (7)	0.0031 (8)
O2	0.0362 (7)	0.0510 (9)	0.0452 (8)	0.0079 (6)	0.0179 (7)	−0.0002 (7)

### Geometric parameters (Å, º)

**Table:**

C1—C2	1.373 (3)	C14—H14	0.9300
C1—C10	1.441 (2)	C15—C16	1.410 (3)
C1—C11	1.532 (2)	C15—H15	0.9300
C2—O1	1.379 (2)	C16—C17	1.411 (3)
C2—C3	1.405 (3)	C16—C21	1.428 (2)
C3—C4	1.353 (3)	C17—C18	1.360 (3)
C3—H3	0.9300	C17—H17	0.9300
C4—C5	1.410 (3)	C18—C19	1.399 (3)
C4—H4	0.9300	C18—H18	0.9300
C5—C6	1.416 (3)	C19—C20	1.369 (3)
C5—C10	1.432 (3)	C19—H19	0.9300
C6—C7	1.351 (3)	C20—C21	1.418 (3)
C6—H6	0.9300	C20—H20	0.9300
C7—C8	1.400 (3)	C22—C23	1.386 (2)
C7—H7	0.9300	C22—C27	1.392 (2)
C8—C9	1.365 (3)	C23—C24	1.395 (3)
C8—H8	0.9300	C23—H23	0.9300
C9—C10	1.418 (3)	C24—C25	1.375 (3)
C9—H9	0.9300	C24—H24	0.9300
C11—C22	1.538 (2)	C25—C26	1.377 (3)
C11—C12	1.546 (2)	C25—H25	0.9300
C11—H11	0.9800	C26—C27	1.383 (3)
C12—C13	1.383 (3)	C26—H26	0.9300
C12—C21	1.441 (2)	C27—H27	0.9300
C13—O2	1.372 (2)	O1—H1	0.9272
C13—C14	1.415 (3)	O2—H2	0.8564
C14—C15	1.353 (3)		
			
C2—C1—C10	117.24 (16)	C15—C14—H14	119.5
C2—C1—C11	121.17 (16)	C13—C14—H14	119.5
C10—C1—C11	121.58 (15)	C14—C15—C16	120.43 (18)
C1—C2—O1	117.96 (17)	C14—C15—H15	119.8
C1—C2—C3	123.37 (19)	C16—C15—H15	119.8
O1—C2—C3	118.66 (17)	C15—C16—C17	121.06 (18)
C4—C3—C2	119.7 (2)	C15—C16—C21	119.10 (17)
C4—C3—H3	120.1	C17—C16—C21	119.83 (19)
C2—C3—H3	120.1	C18—C17—C16	121.39 (19)
C3—C4—C5	120.69 (19)	C18—C17—H17	119.3
C3—C4—H4	119.7	C16—C17—H17	119.3
C5—C4—H4	119.7	C17—C18—C19	119.35 (19)
C4—C5—C6	120.9 (2)	C17—C18—H18	120.3
C4—C5—C10	119.55 (19)	C19—C18—H18	120.3
C6—C5—C10	119.6 (2)	C20—C19—C18	121.0 (2)
C7—C6—C5	121.5 (2)	C20—C19—H19	119.5
C7—C6—H6	119.2	C18—C19—H19	119.5
C5—C6—H6	119.2	C19—C20—C21	121.51 (18)
C6—C7—C8	119.6 (2)	C19—C20—H20	119.2
C6—C7—H7	120.2	C21—C20—H20	119.2
C8—C7—H7	120.2	C20—C21—C16	116.85 (17)
C9—C8—C7	120.7 (2)	C20—C21—C12	123.08 (16)
C9—C8—H8	119.6	C16—C21—C12	120.08 (17)
C7—C8—H8	119.6	C23—C22—C27	117.70 (17)
C8—C9—C10	121.91 (19)	C23—C22—C11	122.13 (16)
C8—C9—H9	119.0	C27—C22—C11	119.94 (16)
C10—C9—H9	119.0	C22—C23—C24	120.67 (18)
C9—C10—C5	116.57 (17)	C22—C23—H23	119.7
C9—C10—C1	124.20 (16)	C24—C23—H23	119.7
C5—C10—C1	119.23 (17)	C25—C24—C23	120.60 (19)
C1—C11—C22	113.67 (14)	C25—C24—H24	119.7
C1—C11—C12	115.99 (14)	C23—C24—H24	119.7
C22—C11—C12	114.36 (14)	C24—C25—C26	119.39 (19)
C1—C11—H11	103.6	C24—C25—H25	120.3
C22—C11—H11	103.6	C26—C25—H25	120.3
C12—C11—H11	103.6	C25—C26—C27	120.10 (19)
C13—C12—C21	117.47 (16)	C25—C26—H26	120.0
C13—C12—C11	124.31 (16)	C27—C26—H26	120.0
C21—C12—C11	117.99 (15)	C26—C27—C22	121.53 (18)
O2—C13—C12	124.56 (17)	C26—C27—H27	119.2
O2—C13—C14	113.82 (16)	C22—C27—H27	119.2
C12—C13—C14	121.56 (18)	C2—O1—H1	100.1
C15—C14—C13	121.02 (18)	C13—O2—H2	100.5

### Hydrogen-bond geometry (Å, º)

Cg is the centroid of the C22–C27 ring.

**Table:**

*D*—H···*A*	*D*—H	H···*A*	*D*···*A*	*D*—H···*A*
O1—H1···O2^i^	0.93	2.49	3.335 (2)	151
O2—H2···O1	0.86	1.85	2.691 (2)	165
C19—H19···*Cg*^ii^	0.93	2.71	3.478 (3)	140

Symmetry codes: (i) −*x*+1, −*y*, −*z*+2; (ii) −*x*, −*y*, −*z*+2.
